# MeQTL analysis of childhood obesity links epigenetics with a risk SNP rs17782313 near MC4R from meta-analysis

**DOI:** 10.18632/oncotarget.13742

**Published:** 2016-12-01

**Authors:** Yuping Tang, Bo Jin, Lingling Zhou, Weifeng Lu

**Affiliations:** ^1^ Department of Orthopaedic, Children's Hospital of Nanjing Medical University, Nanjing, China; ^2^ Department of Neurology, Children's Hospital of Nanjing Medical University, Nanjing, China; ^3^ Surgical Intensive Care Unite, Children's Hospital of Nanjing Medical University, Nanjing, China

**Keywords:** rs17782313, MC4R, meQTL, eQTL, childhood obesitys

## Abstract

Earlier GWAS has identified that rs17782313 near *MC4R* was associated with obesity. However, subsequent studies showed conflicting results, especially among childhood. Besides, the mechanisms underlying the association between rs17782313 and childhood obesity remain largely unexplored, and genetic and epigenetic may interact and together affect the development of childhood obesity. We conducted a comprehensive meta-analysis to assess the association between rs17782313 and childhood obesity. MeQTL and eQTL analysis was applied to explore the effect of rs17782313 on DNA methylation and *MC4R* expression. We found that rs17782313 near *MC4R* was associated with increased childhood obesity risk and BMI z-score in several inheritable models (*P* < 0.05). Additionally, the similar trend was observed among subgroups of Asians, Caucasian. Furthermore, meQTL and eQTL analysis indicated that individuals carrying rs17782313 TT genotype were significantly associated with increased methylation level of cg10097150 located in *MC4R* promoter and decreased expression of *MC4R* than those with CT/CC genotype (*P* = 1.7 × 10^−4^ and *P* = 1.9 × 10^−3^ respectively). Our results strongly confirmed that rs17782313 was associated with increased risk of childhood obesity. Furthermore, rs17782313 T allele was correlated with promoter hypermethylation and decreased expression of *MC4R*, thus involved in the development of childhood obesity.

## INTRODUCTION

Globally, obesity is becoming an increasingly serious clinical and public health challenge and is a major risk factor for various chronic diseases, such as cardiovascular diseases, type 2 diabetes, metabolic syndrome and several types of cancers [1, 2]. The prevalence of obesity is up to 25% in western country and increasing in children [3, 4]. Emerging evidence suggests that childhood obesity resulted in an increased mortality in adults [5]. Environmental factors, especially lifestyle and excessive food intake, are closely related to epidemic obesity. However, multiple twin studies have suggested that genetic factors explain about 65% of the variance in obesity [6, 7]. Genome-wide association studies (GWASs) have identified a number of loci associated with body mass index (BMI), which is a conventional measure of obesity [8, 9]. However, the biological functions of most well known genetic association with small effect size have not fully explained. The majority of GWAS loci are located within non-coding or intergenic regions and do not alter gene coding sequence [10, 11]. It is assumed that GWAS polymorphisms are located in regulatory elements and influence the disease by changing gene expression, which is called expression quantitative trait loci (eQTL) [12, 13].

Recently, it is becoming distinct that epigenetics, especial DNA methylation, also play a key role in obesity pathogenesis. Mounting evidences showed that the SNPs can affect methylation level at nearby CpG sites [14]. Such SNPs are known as methylation quantitative trait loci (meQTLs). The identification of meQTLs may assist in identifying novel candidate disease-associated genes and providing a new approach for connecting DNA sequence variation with phenotype [10].

Previous GWAS revealed that rs17782313 near the melanocortin 4 receptor (*MC4R*) gene was associated with increased risk of obesity in Europeans [15]. It is well established that MC4R plays a key role in coordinating energy homeostasis and body weight [16]. However, subsequent studies from different age groups have showed conflicting and inconclusive results, especially among childhood. Meanwhile, little is known of the molecular biological mechanisms underlying the effect of rs17782313 on the development of childhood obesity.

In this study, we applied a comprehensive meta-analysis to make an accurate assessment of the association of the *MC4R* rs17782313 with childhood obesity risk. Furthermore, most previous studies have investigated genetic and epigenetic mechanisms independently and their interaction was not considered. Our study aimed to combine genetic and epigenetic together to investigate the mechanisms that rs17782313 involved in the development of childhood obesity by using public Gene Expression Omnibus (GEO) and The Cancer Genome Atlas (TCGA) databases.

## RESULTS

### Meta-analysis result

**Figure 1:**
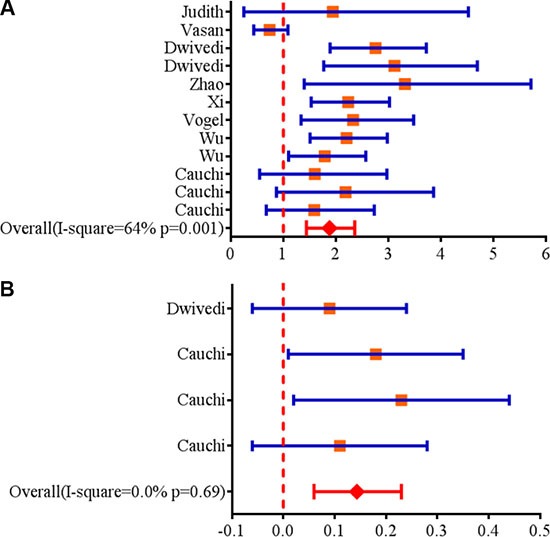
(**A**) Forest plots for the association between *MC4R* rs17782313 polymorphism and genetic susceptibility to childhood obesity (CC vs. TT). Squares boxes indicate the odds ratios and the size of the box is proportional to the weight of the study. Dashed vertical lines represent the null value (OR = 1.0) . Horizontal lines represent the 95% confidence intervals. Diamonds indicate the overall summary estimate derived from a random-effects (RE) model. (**B**) Forest plots for the association between *MC4R* rs17782313 polymorphism and genetic susceptibility to BMI z-score (CC vs. TT). Squares boxes indicate WMD (weighted mean difference) and the size of the box is proportional to the weight of the study. Dashed vertical lines represent the null value (WMD = 0). Horizontal lines represent the 95% confidence intervals. Diamonds indicate the overall summary estimate derived from a fixed-effects (FE) model.

A total of twelve articles including 5,908 cases of obesity and 19,826 controls were enrolled in our meta-analysis study. The detailed characteristics of studies included in this meta-analysis study are supplied in Table 1. The result showed that rs17782313 was significantly associated with childhood obesity in all inheritable models (homozygous model: OR = 1.84, 95%CI = 1.44–2.36; heterozygous model: OR = 1.48, 95%CI = 1.29–1.70; dominant model: OR = 1.35, 95%CI = 1.19–1.55; recessive model: OR = 1.67, 95%CI = 1.39–1.99) (Table 2; Figure 1A). In the stratification analysis, an obvious association was observed between rs17782313 polymorphism and childhood obesity in subgroup of Caucasian and Asian in all inheritable models (*P* < 0.05). Moreover, the similar significant results were also found among subgroups of Taqman and other genotyping methods and sample size more than 1000, but not in the subgroup of sample size less than 1000 under the heterozygous model and recessive model (Table 2). Besides, we applied meta-analysis to evaluate the relationship between rs17782313 genotype and BMI z-score, and four articles were enrolled in the statistical analysis. The results indicated that rs17782313 T > C polymorphism was notably associated with childhood BMI z-score (WMD = 0.14, 95%CI = 0.06–0.23) (Figure 1B). In addition, we obtained a boundary significant result in the replication of public GEO (GSE73103) database with limited samples (dominant model: OR = 1.24, 95%CI = 1.01–1.62, *P* = 0.049).

**Table 1:** Main characteristics of studies included in this Meta-analysis study

Study	Publication year	Country	Ethnicity	Genotyping method	Sample size	Cases	Controls
Judith	2014	Chilean	Mixed	TaqMan	< 1000	238	139
Vasan	2013	India	Asian	TaqMan	≥ 1000	175	998
Dwivedi	2013	India	Asian	iPLEX	≥ 1000	425	904
Dwivedi	2013	India	Asian	iPLEX	≥ 1000	157	904
Zhao	2013	China	Asian	TaqMan	< 1000	371	394
Xi	2011	China	Asian	TaqMan	≥ 1000	1,229	1,619
Vogel	2011	America	Caucasian	TaqMan	≥ 1000	881	434
Wu	2010	China	Asian	TaqMan	≥ 1000	1,207	1,589
Wu	2010	China	Asian	TaqMan	≥ 1000	648	1,589
Cauchi	2009	Finnish	Caucasian	TaqMan	≥ 1000	148	3,802
Cauchi	2009	Finnish	Caucasian	TaqMan	≥ 1000	177	3,392
Cauchi	2009	Finnish	Caucasian	TaqMan	≥ 1000	252	4,062

**Table 2:** Meta-analysis of association between *MC4R* polymorphism and childhood obesity risk

Variables	*n*	CC *vs.* TT		CT *vs.* TT		CC *vs.* TT/CT		CT/CC *vs*. TT	
		OR (95% CI)	*P_het_*	OR (95% CI)	*P_het_*	OR (95% CI)	*P_het_*	OR (95% CI)	*P_het_*
Total	12	1.84 (1.44–2.36)	0.001	1.48 (1.29–1.70)	0.756	1.67 (1.39–1.99)	0.082	1.35 (1.19–1.55)	0.000
Ethnicity									
Asian	7	1.95 (1.39–2.74)	0.000	1.52 (1.30–1.77)	0.561	1.71 (1.34–2.18)	0.015	1.38 (1.13–1.68)	0.000
Caucasian	4	1.80 (1.31–2.46)	0.611	1.38 (1.00–1.90)	0.687	1.61 (1.17–2.20)	0.682	1.33 (1.14–1.55)	0.319
Mixed	1	1.06 (0.25–4.52)	-	0.72 (0.16–3.25)	-	0.97 (0.23–4.13)	-	1.43 (0.87–2.35)	-
Genotyping method									
TaqMan	10	1.66 (1.26–2.19)	0.007	1.49 (1.27–1.75)	0.586	1.58 (1.26–1.99)	0.055	1.26 (1.13–1.41)	0.025
Others	2	2.72 (2.06–3.59)	0.784	1.46 (1.13–1.89)	0.926	1.92 (1.51–2.45)	0.859	2.07(1.68–2.54)	0.776
Sample size									
≥ 1000	10	1.81 (1.39–2.36)	0.001	1.47 (1.27–1.69)	0.812	1.64 (1.36–1.97)	0.068	1.35 (1.16–1.57)	0.000
< 1000	2	2.36 (1.26–4.43)	0.232	1.86 (0.98–3.53)	0.178	1.97 (0.83–4.69)	0.230	1.39 (1.08–1.78)	0.904

### The impact of rs17782313 on *MC4R* expression

**Figure 2:**
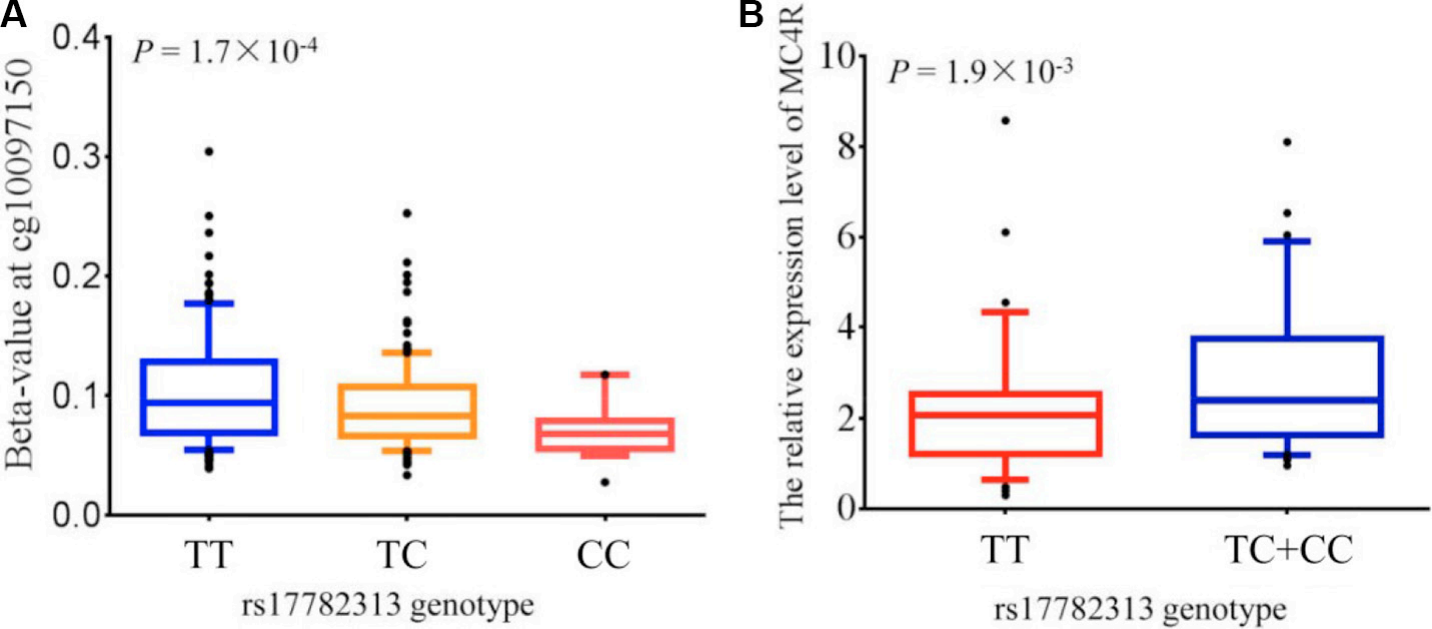
meQTL (methylation quantitative trait loci) and eQTLs (expression quantitative trait loci) analysis for MC4R (**A**) The association between genotype at rs17782313 and methylation levels at the significant CpG probe cg10097150 which is located in the promoter region of *MC4R*. The level of methylation at the CpG probe is shown as the β-value. The box plots show the distribution of the methylation levels in each genotype category with error bars representing the 25% and 75% quantiles. (**B**) *MC4R* expression in normal intestinal tissues stratified by genotype at rs17782313. The box plots show the distribution of the *MC4R* expression levels in each genotype category with error bars representing the 25% and 75% quantiles.

To investigate eQTL, we analyzed data from The Cancer Genome Atlas (TCGA) for whom both germline genotype from Affymetrix Genome-Wide Human SNP Array 6.0 and mRNA expression in intestines normal tissues from Illumina HiSeq 2000 RNA Sequencing Version 2 were available. ANOVA result revealed that individuals carrying rs17782313 TT genotypes were associated with decreased the expression of *MC4R* than those with CT/CC genotypes (*P* = 1.9 × 10^−3^). (Figure 2).

### Association between rs17782313 polymorphism with DNA methylation

Genotype data and correspondent methylation level data were available, which were requested from public GEO database (GSE73103). We extract CpG methylation probes located in 500 kb upstream and downstream from rs17782313. Associations between rs17782313 and methylation levels were tested using a linear model adjusted for age, sex, weight category and a proxy for blood cell type counts. Interestingly, we found that rs17782313 was significantly correlated with methylation level of cg10097150 which is located in the promoter of *MC4R* (*P* = 1.7 × 10^−4^). Here, Individuals with the rs17782313 TT genotype have an obvious hypermethylation level in *MC4R* promoter than those with CT/CC genotype (Figure 2).

## DISCUSSION

Recently, the GWAS have identified rs17782313 was associated with BMI and obesity in Caucasians. However, the relationship of rs17782313 with obesity among other ethnicities are not fully explored, especially in children. In this study, the results of meta-analysis strongly confirmed that rs17782313 near *MC4R* gene was significantly associated with increased risk of childhood obesity in four inheritable models (homozygous model: OR = 1.84, 95%CI = 1.44–2.36; heterozygous model: OR = 1.48, 95%CI = 1.29–1.70; dominant model: OR = 1.35, 95%CI = 1.19–1.55; recessive model: OR = 1.67, 95%CI = 1.39–1.99). Further stratification analyses showed that the similar trend association was also observed in subgroup of Asians, Caucasian, sample size more than 1000. Besides, *MC4R* rs17782313 was boundary correlated with childhood obesity in the replication of available public GEO database (dominant model: OR = 1.24, 95%CI = 1.01–1.62, *P* = 0.049). In addition, another meta-analysis indicated that rs17782313 T > C polymorphism was notably associated with childhood BMI z-score (WMD = 0.14, 95%CI = 0.06–0.23).

SNP rs17782313 mapped 188kb downstream of *MC4R*, which is well established as a candidate gene for obesity. *MC4R* encoded a membrane-bound receptor and member of the melanocortin receptor family protein. Multiple lines of studies suggested that *MC4R* might be involved in regulating energy homeostasis. Activation of *MC4R* can decrease food intake and increase energy expenditure, thereby leading to reduce fat stores [17]. The *MC4R* signaling was identified to regulate the sympathetic nerve activity and glucose tolerance [18]. In addition, the SNPs in *MC4R* play an important role in the susceptibility to obesity. Rare functional mutations in *MC4R* are known to develop childhood-onset obesity [19–22]. Furthermore, mutations leading to complete loss of function are correlated with a more severe phenotype [23], and targeted disruption of the melanocortin-4 receptor also results in obesity in mice [24]. One study has revealed that common genetic variations near or in the MC4R contributes to obesity in American Indians [25]. However, most of GWAS loci are common variants, which located in intergenic and non-coding regions and do not alter change coding sequence [26, 27]. Earlier studies showed that GWAS-identified genetic risk for complex traits often affect phenotype by changing the mount of protein production, rather than altering the type of protein production [28]. We hypothesis the common SNP rs17782313 may influence transcriptional level of *MC4R*, even though the association signal located 188 kb downstream of the *MC4R* coding sequence.

*MC4R* expression are available from public TCGA database, and we obtained evidence that rs17782313 was notably associated with the expression level of *MC4R*.Our result showed that the expression of *MC4R* in individuals carrying rs17782313 TT genotype are significantly lower than that in CT/CC genotype individuals (*P* = 1.9 × 10^−3^).

Given lack of direct functional evidence relating rs17782313 to *MC4R* expression, we assume it alter the methylation level contributing to gene expression. Epigenetic, particular DNA methylation, is a crucial mechanisms of transcriptional regulation that occur without an alteration of the involving DNA sequence [29, 30]. Previous study indicated that gene silencing in cancer are associated with promoter Hypermethylation [31], and strongly correlations were observed between many obesity-associated SNPs and DNA methylation alternation at proximal promoters and enhancers [32]. It was reported that genotype-specific methylation in the promoter of *CHRNB4* affected by SNPs that were remarkably corrected with lung cancer risk [33]. Another study found that rs11636753 G allele was significantly associated with decreased *CHRNA5* DNA methylation, lower *CHRNA5* expression and increased the risk of nicotine dependence [34].

In this study, we apply a method of integrated experiments to explore the effect of common genetic variant rs17782313 on DNA methylation from available GEO database. Interestingly, linear regression revealed the prominent association between rs17782313 and methylation level of cg10097150 which is located in the promoter of *MC4R* (*P* = 1.7 × 10^−4^). Or rather, individuals carrying rs17782313 TT have a remarkably hypermethylation level in *MC4R* promoter than those with CT/CC genotype. Altogether, our results provide evidence that rs17782313 may affect the methylation level of *MC4R*, which contribute to regulate *MC4R* expression, thus involved in the development of childhood obesity.

There are some limitations in our study. First, our analysis is based on public database and studies with greater sample size are needed to validate our findings.

Additionally, the interaction between environmental exposures and genetic and epigenetic mechanisms should be considered in the following research.

In summary, our meta-analysis of data from current and published studies confirms the significant association between *MC4R* polymorphism and the risk of childhood obesity and BMI z-score. Furthermore, eQTL and meQTL results strongly indicate that obesity-associated rs17782313 T allele was significantly associated with promoter hypermethylation and decreased expression of *MC4R*, thus involved in the childhood obesity. This study highlights that genetic and epigenetic mechanism in *MC4R* promoter may interact and together affect functional relevance for *MC4R* expression and development of childhood obesity.

## MATERIALS AND METHODS

### Literature and search strategy

We search relevant articles published in English from following databases, including PubMed and Embase. The search strategy to identify all possible studies involved the use of the following keywords: (*MC4R*) and (polymorphism OR variant OR genotype) and (obesity). References of the retrieved articles were hand-searched.

### Inclusion criteria and data extraction

The enrolled studies met the following inclusion criteria: (1) Case-control, cross-sectional, cohort studies which evaluated the association of *MC4R* rs17782313 with obesity. Cases diagnosed by the WHO standards of childhood obesity patients. Height-standard weight method was applied among age less than 10-year-old and BMI (Body mass index) was used for 10 ~18 years old children to assess obesity levels. The control group was composed of normal healthy children; (2) Providing an odds ratio (OR) with 95% confidence interval under various inheritable models or sufficient raw data to calculate these estimates; (3) Reliable and high-quality data by using appropriate statistical analysis method; (4) If data were repetitively reported, the study with biggest sample size was included. Two authors independently assessed the articles for compliance with the inclusion criteria, and inconsistent were resolved by agreeing with the third reviewer.

### The cancer genome atlas (TCGA) and gene expression omnibus (GEO) databases

Gene expression profiles were downloaded from TCGA project by RNA-Seq (level 3). In total, 434 colon adenocarcinoma tissues and 41 normal colon samples were included. To control for potential batch effects of mRNA expressions, a series of normalizations and corrections were applied. Briefly, level 3 mRNA expression of each gene was log2 transformed if it was not normally distributed, and genes with zero values were removed. We also accessed TCGA individual level 2 SNP data from tissues and blood, which were genotyped with an Affymetrix Human Genome Wide SNP 6.0 array. The genotype of rs17782313 and corresponding methylation levels were available from GEO database (GSE73103). A total of 355 healthy individuals were genotyped and obtained DNA methylation levels in their blood using the Illuminal 450K BeadChip.

### Statistical analysis

Meta-analysis was conducted by STATA version 11 (StataCorp LP, College Station, TX, USA). Heterogeneity was evaluated by Cochran'Q and *I*^2^ statistics. *P*_het_ ≤ 0.10 and *I*^2^ values > 75% were considered to be significant heterogeneity and we used random-effect model to calculate combined effect, otherwise we applied fixed-effect model. *P* < 0.05 was used as threshold to exclude genotype data which did not meet Hardy-Weinberg equivalent. Data were pooled across studies, and OR (odds ratio) and 95%CI (confidence interval) for categorical variables and WMD (weighted mean difference) for continuous outcomes, weighted according to study sample size, were calculated. We applied linear regression to evaluate the relationship between rs17782313 risk allele and gene epigenetic and expression alterations from public GEO and TCGA database by using R software. All statistical analyses were two-sided, and a *P value* < 0.05 was considered statistically significant.
